# Occupational hearing loss in teachers: a probable diagnosis.

**DOI:** 10.1016/S1808-8694(15)31072-7

**Published:** 2015-10-22

**Authors:** Regina Helena Garcia Martins, Elaine Lara Mendes Tavares, Arlindo C. Lima Neto, Marisa P. Fioravanti

**Affiliations:** 1 Assistant Professor, Doctor on Surgery at the Botucatu Medical School - Unesp. Head of the phoniatrics and voice outpatient unit. Teacher of Otorhinolaryngology at the Paulista State University - Unesp, Botucatu campus.; 2 Master's degree at the Botucatu Medical School. Speech therapist.; 3 Resident in the Otorhinolaryngology department, Unesp, Botucatu.; 4 Post-graduate student at the Botucatu Medical School - Unesp. Speech therapist in the Otorhinolaryngology discipline of the Botucatu Medical School - Unesp. Júlio de Mesquita Filho Paulista State University - Unesp, Botucatu campus.

**Keywords:** hearing loss, teacher, noise

## Summary

Teachers frequently report auditory symptoms and excessive noise in classrooms, but noise level measurements are not done routinely. Study model - a prospective clinical trial. **Aim** - To study auditory symptoms and audiometric exams of teachers and classroom noise levels. **Material and Method:** Data from two groups, GI (40 teachers) and GII (40 voluntaries) were studied as follows: age, gender, working conditions, audiometric exams, and classroom noise levels. **Results** - In GI there were more females (86%), working in basic teaching (75%), in classes with 21-40 students (70%), with workloads between 26 and 40 hours per week (47%), and variable professional teaching time. Most teachers in GI reported excessive classroom noise (93.5%) and auditory symptoms (65%). In GI, 25% of teachers presented audiometric alterations (versus 10% of controls), with an acoustic notch predominating (11.25%; p<0.05). Noise levels close to 87dBA were recorded in classes at all teaching levels. **Conclusions** - occupational hearing loss may occur in teachers. Further studies are needed to confirm this proposition.

## INTRODUCTION

Noise-induced occupational hearing loss has been a long-time concern of health professionals. Research on this condition has focused mostly factory and manufacturing workers.^1^, ^2^, ^3^, ^4^ Less attention, however, has been given to teachers exposed to classroom noise. It is known that excessive noise in classrooms with many pupils not only hinders learning, but may also lead to psychological harm and organic damage in teachers. These professionals frequently complain of hearing loss, vestibular conditions, tinnitus, and extra-auditory symptoms such as irritability, sleeping difficulties, digestive problems, behavioral disorders, concentration difficulties, and others.^5^, ^6^, ^7^

An investigation of noise-induced hearing loss in teachers would require confirmation of excessive noise levels in work environments by measuring classroom noise during classes. Pereira et al.^8^ did these measurements in classes where 12 elementary school teachers in a single public school worked; they found a maximum noise peak of 86dBA and a minimum noise peak of 52dBA.

The audiometric configuration of noise-induced hearing loss shows symmetrical, mild to moderate sensorineural hearing loss mostly at 3,000 and 4,000 Hz.^9^, ^10^, ^11^ Audiometry should be done upon admission (the initial reference test) to define occupational hearing loss in professionals exposed to noise, and repeated after 6 months and annually. The initial audiometric test is compared to subsequent tests to investigate hearing loss, defined as a 10 Hz or more difference in auditory thresholds at 3,000, 4,000, and 6,000 Hz or worsening of 15 Hz or more in at least one of these frequencies. 9, 10, 11

Most authors believe that an individual may develop noise-induced hearing loss if exposed to constant or intermittent noise at 85dB during at least eight hours of work a day.^9^, ^10^, ^11^ Many teachers work longer hours in classrooms, including night classes. Routine classroom noise measurements are not done routinely, and there are few papers showing the results of the measurements that are done. It is, therefore, impossible to establish with any precision the true causes of hearing loss in teachers. The situation is different among industry workers, where environmental noise is periodically measured, and where workers are required to use protection equipment.

These comments show the need for evaluating in greater depth the work environment of teachers to identify factors that affect hearing in these professionals.

## OBJECTIVES

Participants in this study included public and private school teachers and aimed to:

• identify auditory symptoms;
• assess auditory acuity;
• verify classroom noise levels;
• correlate audiometric test results to noise exposure;
• compare audiometric test results of teachers and of a volunteer control group.

## SERIES AND METHOD

### Series

The study was approved by the Botucatu Medical School Research Ethics Committee for investigation in human beings (Document number 113/2003). All participants signed a free informed consent form. The series included a sample group (GI) that included 80 teachers from 10 public and private schools in the city of (Sao Paulo state). A control group (GII) was also included to facilitate the interpretation of auditory test results. This group was composed of 40 similarly aged non-teacher volunteers to which were applied the same exclusion criteria.

**Figure 1:**
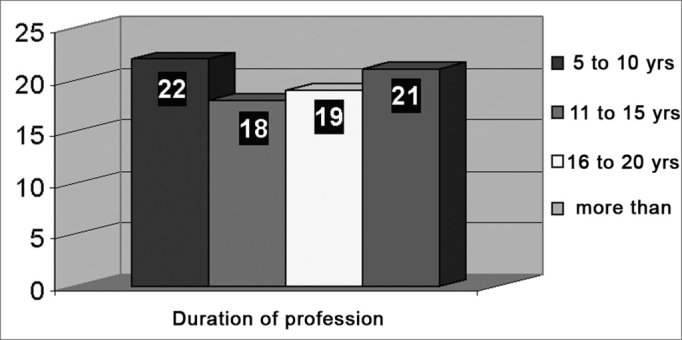
Distribution of teachers according to duration of professional work.

The sample group (GI) included 69 women (86%) and 11 men (14%). The control group included 67.5% women and 32.5% men. The age range of teachers was 24 to 59 years (mean = 40.2 years), concentrating on the 36 to 50 year age group (55%). The duration of professional activities was uniform throughout the sample group (Figure 1).

The weekly workload was as follows: 20 teachers (25%) worked less than 25 hours, 38 teachers (47.5%) worked between 26 and 40 hours, and 22 (27.5%) worked more than 40 hours. There were 60 elementary school teachers (75%), 13 pre-school teachers (16%), and 7 junior school teachers (9%). There were 21 to 40 students per classroom for 56 teachers (70%); there were fewer than 20 students per classroom for 10 teachers (12.5%); there were over 40 students per classroom for 14 teachers (17,5%).

Exclusion criteria were: a history of otorrhea or otological surgery; congenital or family deafness; auditory pathway malformation; current or past work in another activity where there is/was exposure to excessive noise; use of ototoxic drugs; users of individual sound amplification devices; a history of cranial trauma; altered immitance tests; and work in special subjects (physical education, teaching of religion, teaching foreign languages, etc.). Inclusion criteria were: work as a teacher for over five years; a minimum weekly workload of 20 hours in curricular subjects.

### Methodology

Protocol - teachers initially were interviewed to obtain protocol data on age, gender, auditory symptoms, and working conditions (duration of the profession, number of students per classroom, workload, education level taught, and a description of environmental noise).

Assessment of auditory acuity - teachers in the sample group and control group volunteers initially underwent otoscopy. An assessment of auditory acuity was done with appropriately calibrated devices. Tests included pure tone audiometry (Amplaid® A321 audiometer, Italy) and logoaudiometry (speech audiometry). Audiometric results were classified according to Russo & Santos (1993);12 normal results were air and bone auditory thresholds between 0 and 25dBNHL. Hearing loss according to audiometry was classified as: flat, sloping, rising, inverted U, and notches (at least a 30dB reduction of the audiometric threshold at 4,000 and/or 6,000Hz). When audiometric thresholds were 0 or 5 dB, and if there was a 25dB reduction of the audiometric threshold at 4,000 and/or 6,000Hz only, the authors classified the audiometric configuration a tending to notches. Speech therapists working as collaborators did the auditory assessments.

Measurement of environmental noise - an appropriately calibrated decibelimeter (Larson & Davis® model 812) was used. This device was operated by a single trained technician, who measured classroom noise levels in different teaching levels in three situations: during teacher explanations, during student participation, and during classroom discussions. Minimum and maximum measurements were made, and the energy-equivalent noise level (Leq), which is the average of the sound energy during the measurement period using a dB(A) frequency filter.

Statistics - data was analyzed using the chisquared method and the comparison of proportions test based on a normal distribution. The significance level was p values equal to or below 0.05.

## RESULTS

Report of classroom noise - 93.75% of teachers reported excessive classroom noise.

Auditory symptoms - Table 1 shows that 65% of teachers had auditory complaints. The most common symptom was hypoacusis (31.25%), frequently associated with tinnitus and/or vertigo.

**Table 1:** Prevalence of auditory symptoms in teachers.

Teachers		
Symptoms	N	%
No complaints	28	35.00
Hypoacusis	25	31.25
Hypoacusis and tinnitus	6	7.50
Hypoacusis, tinnitus and vertigo	5	6.25
Tinnitus	4	5.00
Vertigo	2	2.50
Pain	1	1.25
Fullness	1	1.25
Tinnitus and fullness	2	2.50
Hypoacusis and pain	1	1.25
Tinnitus and vertigo	2	2.50
Hypoacusis and vertigo	2	2.50
Hypoacusis, Tinnitus and pain	1	1.25
Total	80	100.00

**Figure 2:**
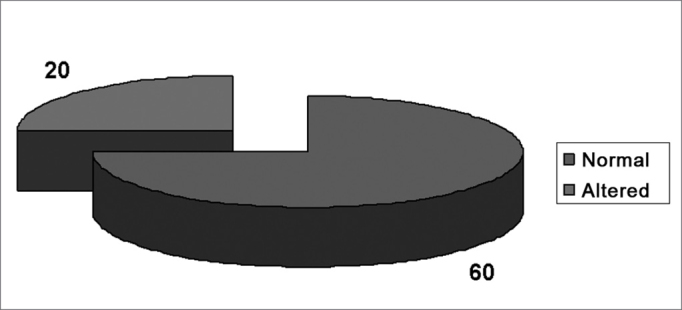
Results of audiometric exams of teachers (GI).

**Figure 3:**
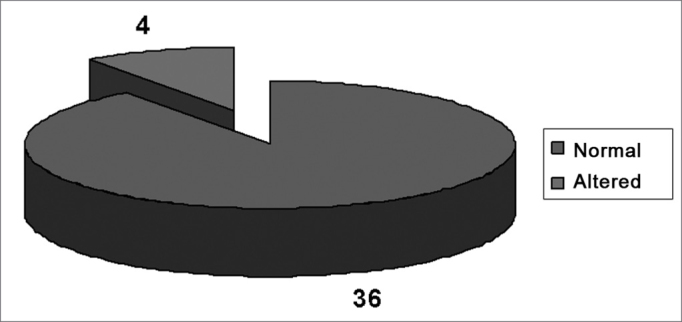
Results of audiometric exams of volunteers (GII).

Results of audiometric tests - audiometric tests (Figures 2 and 3) and audiometric curve configurations (Table 2) revealed that 20 teachers in the sample group (40 ears, 25%) and only four volunteers in the control group (8 ears, 10%) had some degree of hearing loss. Notches were the most frequent audiometric configuration, found in 18 ears of teachers in GI (11.25%) and in one ear of control group volunteers (1.20%; p<0.05). The sloping audiometric curve was also frequent (GI-8.10% and GII-7.50%; p>0.05), followed by a tendency to notches (GI-1.87% and GII-1.20%; p>0.05).

**Table 2:** Audiometric tracing configuration of teachers (GI) and of the control group (GII) according to the number of ears tested.

Audiometric tracing	Sample group		Control group		Value dep
	N	%	N	%	
Normal test	120	75.00	72	90.00	0.003*
Acoustic drop	18	11.25	1	1.20	0.003*
Sloping	13	8.10	6	7.50	0.43
Tendency to notches	3	1.87	1	1.20	0.36
Inverted U	2	1.25	0	0.00	0.16
Horizontal	2	1.25	0	0.00	0.16
Rising	2	1.25	0	0.00	0.16
Total	160	100	80	100	

All of the participants had a type A normal tympanometric curve.

Classroom noise level - maximum and minimum classroom sound pressure level verification values and the Leq are shown on Table 3; significant environmental noise was found at all teaching levels, at around 87.4dB (A), reaching 89dB (A) in junior school.

**Table 3:** Minimum and maximum values and the Leq of classroom noise level verification in different teaching levels (in dBA).

Noise level (dBA)*	Leq B(A)		Leq dB(A)	
School level	Minimum	Maximum	Minimum	Maximum
Basic School	63.7	87.4	73.5	82.5
Elementary School	59.8	87.4	69.6	84.2
Middle School	66.9	89.0	74.6	86.4

* Leq –equivalent noise level.

## DISCUSSION

This study shows that there were more female teachers in the sample group. Many of them had classrooms with many students and worked over 40 hours a week. This workload probably reflects low wages and the need to supplement the family income, as some authors have pointed out.^13^, ^14^

The working environment of teachers should receive more attention, given the time they remain in classrooms. Crowded classrooms generate significant noise and place excessive demands on phonation. In this context noise verification has not been routine or even required. Most of the teachers in this study reported excessive classroom noise. 190 of 240 teachers assessed by Pérez Fernandez & Preciato López^14^, ^15^ made similar complaints in a specific questionnaire.

Based on our study we believe that teachers exposed to classroom noise may develop occupational hearing loss throughout their career, given the significant number of auditory symptoms, frequent reports of excessive classroom noise, a large number of altered audiometries compared to the control group (40 × 18), and high classroom sound pressure values in teachers belonging to the GI group. Many teachers had notches in their audiometric configuration, reinforcing the abovementioned assumption. This curve is typical of noise-induced hearing loss and was found in only 1 control group volunteer and in 18 sample group teachers. This diagnosis, however, is based on a reduction of audiometric thresholds when compared to pre-placement exams, which have not been standardized for teachers. Confirmation, therefore, is limited.

Verification of classroom sound pressure levels is not done routinely, but our investigation revealed elevated values, ranging from 59.8 dB (A) to 89 dB (A). If the acceptable noise level for industry workers is 85dB, and that studies on acoustics applied to education suggest tolerable classroom sound pressures of 40 to 70dB(A),2,16 we conclude that teachers are exposed to excessive noise levels, notwithstanding Bovo & Galceran's17 findings of classroom noise levels up to only 55dB.

The regulating rule number 15 of the Ordinance 3.214/78,18 which defines tolerance levels for exposure to noise, refers to continuous and intermittent noise, such as that in classrooms. Jiang^19^ underlines the harmful effects of noise originating outside classrooms, showing an increased prevalence of hypoacusis at high frequencies in physical education teachers.

Various authors in past decades have tried to demonstrate the harmful effects of exposure to excessive noise on auditory pathways. This includes studies of industry machine operators,^20^,^21^ musicians,^22^ military personnel,^23^ drivers,^24^ and other recently investigated classes such as hospital workers^25^ and neonates in ICUs.^26^ These papers have provided the basis for assuring the health promotion rights of various workers. Few of these studies, however, have focused on teachers, who experience inadequate working conditions and frequent auditory symptoms.

## CONCLUSION

The high frequency of auditory symptoms, constant reports of excessive classroom noise, the detection of a significant percentage of altered exams with a predominance of notches, and verification of high environmental noise levels, suggest that occupational hearing loss due to noise exposure is present in teachers. This diagnosis needs to be confirmed through pre-placement and periodic exams and further careful studies similar to this paper.
